# Characterization of a *PRKCE*::*ETV6* fusion as a potential oncogenic driver in T-cell acute lymphoblastic leukemia

**DOI:** 10.1186/s40348-025-00208-x

**Published:** 2025-10-22

**Authors:** Esther L. Monsees, Udo zur Stadt, Julia Strauss, Sabrina Schuster, Nadja Kleist, Richard T. Hauch, Michael Spohn, Gerrit Wolters-Eisfeld, Martin A. Horstmann, Gabriele Escherich, Lena Behrmann

**Affiliations:** 1 Clinic of Pediatric Hematology and Oncology, University Medical Center Hamburg-Eppendorf, Hamburg, Germany; 2 Research Institute Children’s Cancer Center Hamburg, Hamburg, Germany

**Keywords:** Acute lymphoblastic leukemia, ETP-ALL, Gene–gene fusions, *ETV6::INO80D*, *PRKCE::ETV6*

## Abstract

**Background:**

T-cell acute lymphoblastic leukemia (T-ALL) is an aggressive hematologic malignancy caused by mutation accumulation during hematopoiesis. The characterization of chromosomal abnormalities may provide significant insights into genetic mechanisms of malignant transformation in hematopoietic cells. However, T-ALL is genetically very heterogenous and driving mutations as well as clonal markers for the assessment of minimal residual disease are not always identifiable. Hence, there is a clinical need to further refine the genetic landscape of T-ALL including previously unrecognized fusion partners of commonly translocated genes in T-ALL of childhood.

**Results:**

In this study, we screened *n* = 229 T-ALL cases by our targeted genomic capture high-throughput sequencing (gc-HTS) approach. In total, we identified *n* = 60 gene–gene fusions, present in *n* = 57 (25%) of the patients. Nine rare or even unrecognized translocations were identified and validated. Furthermore, owing to its interesting chromosomal structure, we studied the oncogenic potential of the complex rearrangement of chromosome 2 and 12, found in a near-early T-cell progenitor (ETP) ALL that leads to the fusion events *PRKCE::ETV6* and *ETV6::INO80D*. Exogenous expression of *PRKCE::ETV6* in Ba/F3 pro-B and D1 T-cells caused interleukin-independent proliferation and enhanced survival upon interleukin withdrawal, respectively.

**Conclusion:**

Our study underlines the heterogenous mutational landscape in T-ALL. The previously unrecognized *PRKCE::ETV6* resulting from a complex rearrangement involving chromosome 2 and 12 demonstrated transforming potential in cytokine-dependent cellular models support the notion of a driver mutation in near ETP-ALL. Our data reconfirm the relevance of ETV6-fusion proteins in the pathogenesis of undifferentiated T-ALL. Importantly, genomic breakpoints at the *ETV6* locus represent potentially robust MRD markers for (near) ETP-ALL that lack IG/TR rearrangements.

**Supplementary Information:**

The online version contains supplementary material available at 10.1186/s40348-025-00208-x.

## Background

Acute lymphoblastic leukemia (ALL) is a hematological malignancy of lymphoid progenitor cells, with accumulation of genomic lesions leading to abnormal proliferation [1]. ALL is a heterogenous disease but can be classified by immunophenotyping mainly as B-cell precursor-ALL (BCP-ALL) (85%) or T-cell ALL (T-ALL) (15%). With recent advances in treatment, risk stratification, and in supportive care, the event-free survival rate (EFS) for childhood ALL has increased to > 90% [2]. However, T-ALLs remain challenging and have an unsatisfactory rate of refractory disease and relapses. 20–30% of pediatric T-ALL cases relapse, and the five-year overall survival rate after relapse is less than 20% [3].

On the one hand, T-ALL is characterized by extensive genetic heterogeneity, therefore developing broadly applicable molecular targeted therapies poses significant challenges [4]. On the other hand, paucity of leukemia-specific T-ALL epitopes and T-cell fratricide due to shared expression of targetable antigens on both malignant and healthy T-cells challenge the development of T-cell-based immunotherapies [5]. Consequently, there are fewer available targeted therapies and limited immunotherapeutic options for T-ALL patients than for BCP-ALL [4].

Over the past decades, many genetic abnormalities were identified in T-ALL. Notably, chromosomal translocations of oncogenes (e.g. transcription factors) and the *T-cell receptor* (*TCR*) loci can lead to aberrant expression driven by the *TCR* promoter activity, resulting in dysregulated T-cell differentiation and proliferation [6]. *LMO1/2, TAL1/2,* or *TLX1* are frequently translocated to the *TCR* locus. In addition, recurrent gene–gene fusion events (20–30% in T-ALL) lead to overexpression of wild type proteins (e.g. *STIL::TAL*) [7] or fusion proteins with aberrant protein functions (e.g. *NUP214::ABL1*) [8].

More recently, next-generation sequencing has extended the repertoire of genetic and epigenetic abnormalities in T-ALL uncovering a wide range of heterogenous mutations [9, 10]. Of note, an immunophenotypic subtype of T-ALL (early T-cell progenitor, ETP-ALL) shows a specific gene mutation pattern, marked by mutations within genes regulating cytokine receptors and RAS signaling [11].

In this study, we utilized data from our targeted panel for genomic capture high-throughput sequencing (gc-HTS) that we utilize to select molecular MRD markers to identify novel fusion partners of commonly translocated genes in T-ALL [12]. We screened *n* = 229 cases of T-ALL and identified *n* = 60 gene–gene fusions in 25% of the patients. Among those we identified several gene fusion events that have not been previously described in T-ALL. Notably, a near ETP-ALL sample exhibited a previously unrecognized *ETV6*-fusion gene with transforming potential. Our results indicate that *PRKCE::ETV6* expression induces cytokine-independent growth and survival in Ba/F3 pro-B cells and D1 T-cells compatible with an oncogenic driver mutation in ETP-ALL.

## Results

### Patient characteristics

By use of gc-HTS [13] we screened *n* = 229 pediatric T-ALL patients, diagnosed in the CoALL trial centers in Germany between 2003 and 2023. Sex distribution was unequal as anticipated (f = 65 and m = 173). The average age at diagnosis was 9.4 years (range 1.1–18 years) and mean WBC was 64.3/nl (1–900).

### Gene–gene fusions

**Fig. 1:**
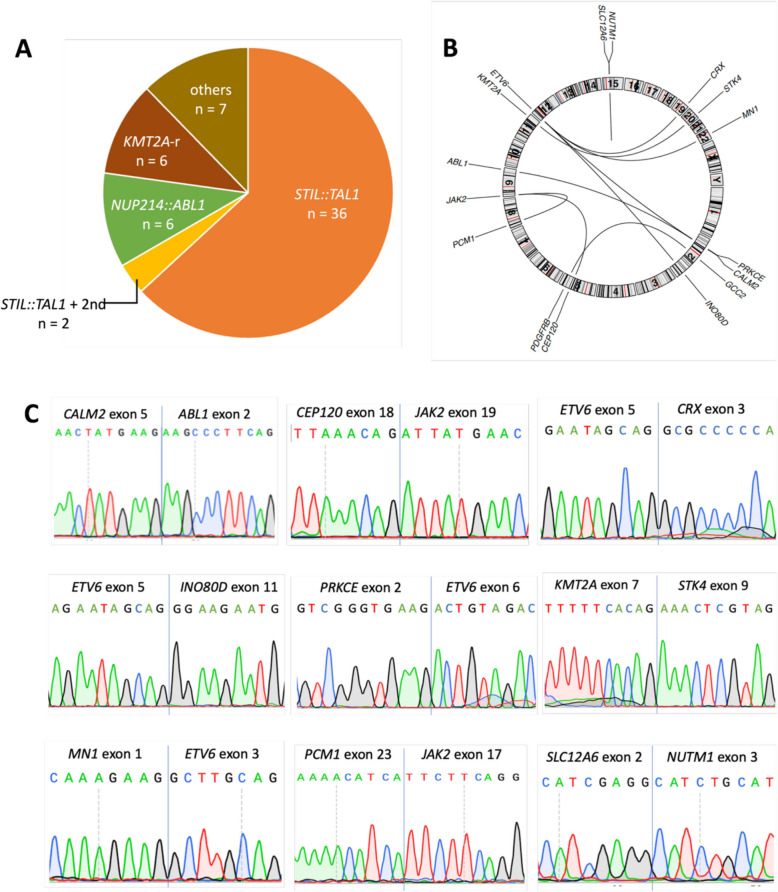
Overview of gene–gene fusions identified in 229 pediatric T-ALL cases in this study. **A** Number of gene–gene fusions identified by targeted gc-HTS in 229 T-ALL cases. **B** Circos plot of 9 rare or previously unknown rearrangements. **C** Validation of genomic breakpoints from detected gene–gene fusion transcripts in cDNA by Sanger sequencing

Within the targeted gc-HTS data we identified *n* = 60 gene–gene fusions in 25% of the patients (*n* = 57 out of 229). Within the gene–gene fusion events, we identified mainly *STIL::TAL1* with a frequency of 17% (38/229), *NUP214*::*ABL1* (3%, 6/229) and *KMT2A*-rearrangements (3%, 6/229). Two patients with *STIL::TAL1* fusions each had an independent second translocation, T-ALL 01 had a t(2;5) and T-ALL 02 had an additional t(8;9) translocation (Table 1, Fig. 1A). Detailed analysis of case T-ALL 01 with the fusion gene *GCC2::PDGFRB* was published elsewhere by us [14]. All other events represent rare or previously unknown events for T-ALL (Fig. 1B). In T-ALL 03, we identified two gene–gene fusions, involving chr. 2 and 12. To independently validate the findings, we performed targeted cDNA sequencing of the fusion sites (Fig. 1C).

**Table 1:** Overview of the findings of gc-HTS analysis in 9 cases with previously unidentified gene–gene fusions in this study cohort. The gc-HTS analysis identified 7 rare and 3 novel gene–gene fusions in the 9 samples analyzed. References indicate a previous identification of the gene–gene fusion in T-ALL or other leukemic entities

Case ID	Fusion	ALL subtype	Chromosome location		Breakpoint 5’ gene	Breakpoint 3’ gene
			**Gene 1**	**Gene 2**		
T-ALL 01	*GCC2::PDGRFB *[14] *(STIL-TAL1)*	Cortical T	2q12.3 (1p32 [del(1p)])	5q32	Intron 16	Exon 9
T-ALL 02	*PCM1::JAK2 *[15] *(STIL::TAL1)*	Pre-T	8p22 (1p32 [del(1p)])	9p24.1	Intron 23	Intron 16
T-ALL 03	*ETV6::INO80D *[11] *PRKCE::ETV6*	Near ETP	12p13.2 2p21	2q33.3 12p13.2	Intron 5 Intron 2	Intron 10 Intron 5
T-ALL 04	*CALM2::ABL1*	pre-T	2p21	9q34.12	Intron 5	Intron 1
T-ALL 05	*CEP120::JAK2*	Cortical T	5q23.2	9p24.1	Intron 18	Exon 17
T-ALL 06	*ETV6::CRX *[16]	Near ETP	12p13.2	19q13.33	Intron 5	Intron 2
T-ALL 07	*SLC12A6::NUTM1*[17]	Cortical T	15q14	15q14	Intron 2	5’UTR
T-ALL 08	*KMT2A::STK4 *[18]	Mature T	11q23.3	20q13.12	Intron 8	Intron 10
T-ALL 09	*MN1::ETV6 *[19]	Near ETP	22q12.1	12p13.2	Intron 1	Intron 2

### Complex rearrangement involving chromosome 2 and 12 results in two gene–gene fusion products

**Fig. 2:**
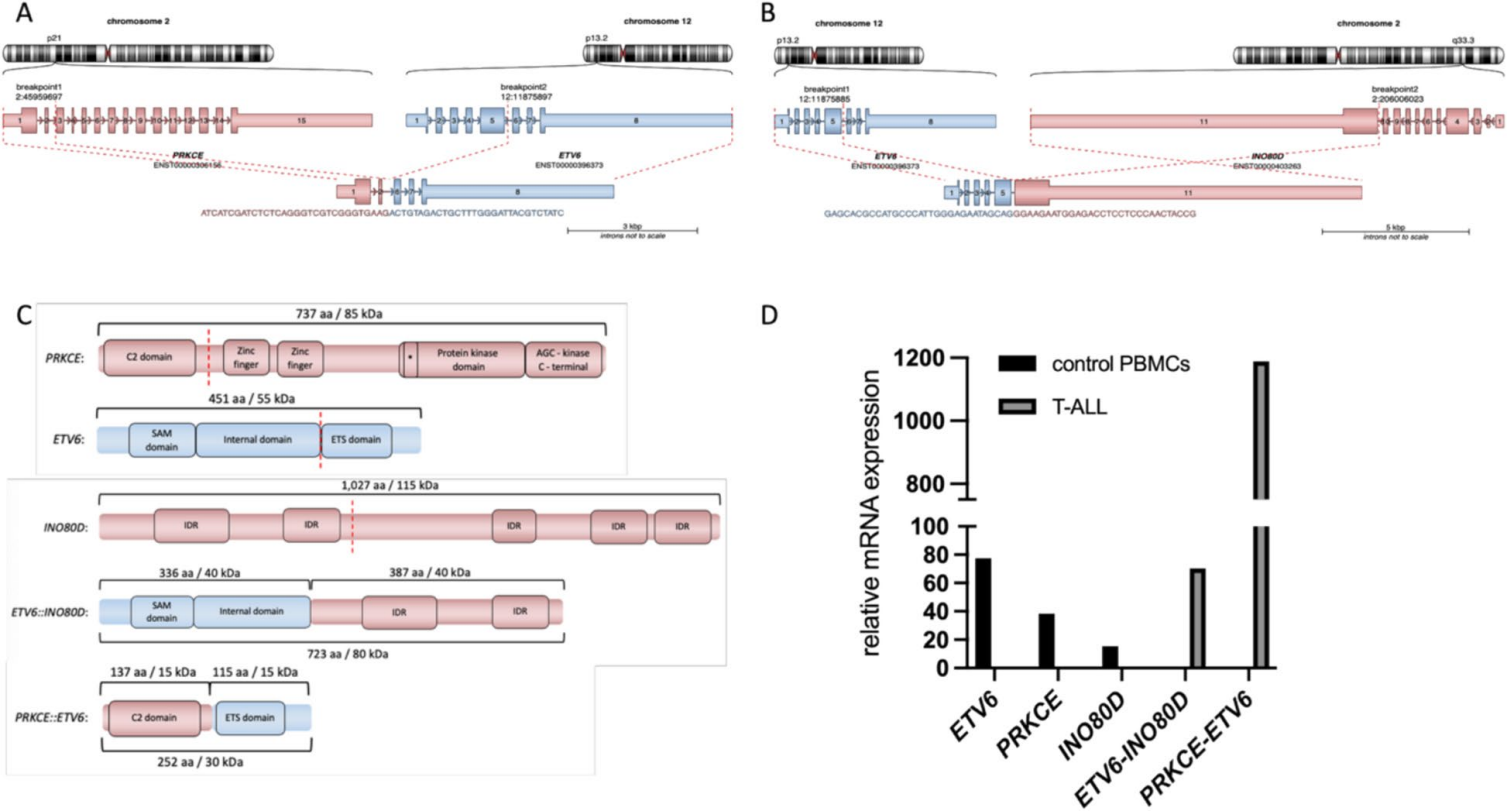
Characterization of *PRKCE::ETV6* and *ETV6::INO80D* fusions identified in a pediatric T-ALL patient: **A** + **B** Chromosomal position and exon structure of *PRKCE, ETV6* and *INO80D*. Dashed lines comprise the exons that occur in the *PRKCE::ETV6* and *ETV6::INO80D* gene fusions. **C** Schematic structure of functional domains from PRKCE::ETV6 and ETV6::INO80D fusion proteins. The number of amino acids and molecular weight (in kDa) are specified. Asterisk marks the ATP-binding site. **D** Relative mRNA expression in patient’s cDNA (T-ALL) and control PBMCs (peripheral blood mononuclear cells) from healthy donors for wildtype *ETV6, PRKCE, INO80D*, and the fusion product *ETV6::INO80D* and *PRKCE::ETV6*. Data show 2^-ΔΔCt*1000, normalized to human B2M. aa, amino acids; chr, chromosome; kbp, kilo basepairs; IDR, intrinsically disordered region; SAM, sterile alpha motif

In regard of the distinct chromosomal breakpoint pattern, we analyzed the T-ALL 03 fusions in detail (gc-HTS identified breakpoints mapped to chr2:45,959,697, chr12:11,875,897, chr12:11,875,775 and chr2:206,006,023 (hg38)). This specific translocation generates gene fusions of *PRKCE* (chr. 2, p21) with *ETV6* (chr. 12, p13.2) and *ETV6* (chr. 12, p13.2) with *INO80D* (chr. 2, q33.3). The schemes of chromosomal translocations and its products are shown in Fig. 2A + B. According to our gc-HTS data, cytogenetic studies obtained from clinical records at the time of diagnosis presented a highly aberrant and complex karyotype of 47,XY,?der(2),del(11)(q14 ~ 22),add(12)(p13), + mar[cp10]/46,XY[7]. FISH analysis at that time showed a deletion at chromosome 11q leading to the loss of one of the *ATM* genes, located in the region 11q22.

The in-frame fusion of *PRKCE::ETV6* produces a 7,233 bp transcript, which is made of the N-terminal 838 bp residues (exon 1–2) of *PRKCE* (ENST00000306156) and the C-terminal 6,395 bp residues (exon 6–8) of *ETV6* (ENST00000396373). *ETV6::INO80D* generates an in-frame fusion of 13,276 bp in total, which is made of the N-terminal 1,463 bp residues (exon 1–5) of *ETV6* and the C-terminal 11,813 bp residues (exon 11) of *INO80D* (ENST00000403263). The *PRKCE* breakpoint is localized in intron 2, which leaves the C2-domain part of the *PRKCE::ETV6* fusion. The ATP-binding site and the protein kinase domain from *PRKCE* are not part of the *PRKCE::ETV6* fusion. *ETV6* breakpoint is located in intron 5, preserving the ETS-domain in the *PRKCE::ETV6* fusion. The reciprocal part of *ETV6* remains integral to the *ETV6::INO80D* fusion, including its SAM-domain. *INO80D* breakpoint is in intron 10 (Fig. 2C). *ETV6::INO80D* has previously been identified in two T-ALL patients described by Zhang et al. [11]. *PRKCE::ETV6* on the other hand, has not been documented in previous studies. Relative expression of fusion transcripts was determined by quantitative real-time PCR (qPCR) (Fig. 2D).

### Clinical presentation of patient with complex rearrangement involving chromosome 2 and 12

**Fig. 3:**
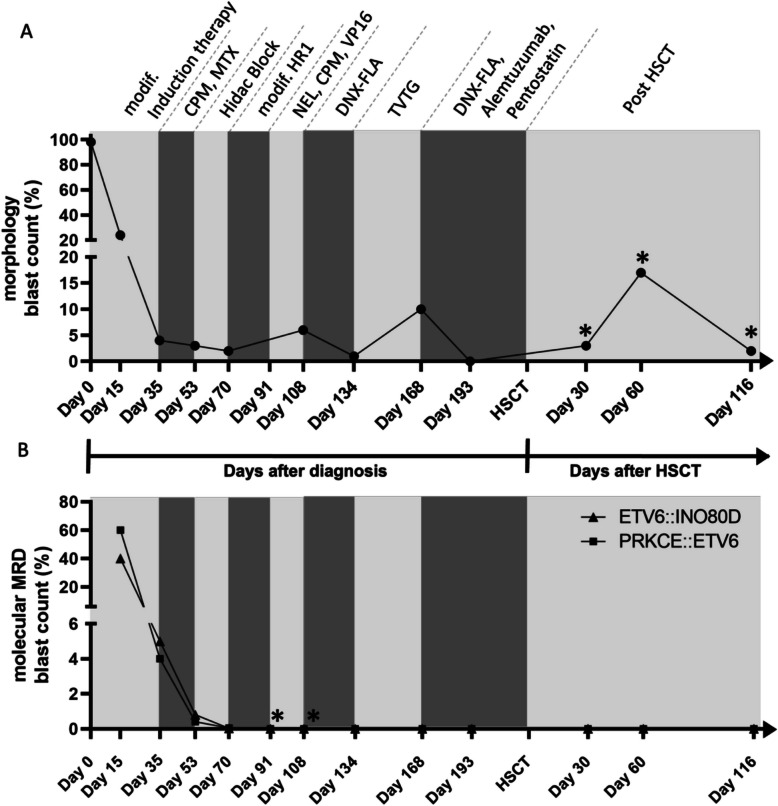
Clinical timeline and treatment response of the T-ALL case *with PRKCE::ETV6* and *ETV6::INO80D*: **A** The blast count by morphology (%) is depicted over the course of the treatment interventions. Induction therapy was initiated with a modified CoALL-08–09 High Risk Standard treatment due to hyperbilirubinemia and hepatopathy of unknown origin. Treatment was started with a Cortisone-monotherapy, later Vincristine/Cyclophosphamid instead of Vincristine/Daunorubicin was added. The therapy was continued with low doses of Daunorubicin, i.th. Methotrexat was given and another two triple therapies had to be administered. Due to inadequate further MRD decrease, it was decided to intensify therapy. Patient received a hidac block, a modified high-risk block and a TVTG block. The treatment was then switched to Alemtuzumab and Pentostatin, under which a stable remission was achieved for the first time. Patient then received a bone marrow transplant with an HLA-mismatched unrelated donor (HLA 9/10). *Reappearance of blast after HSCT was excluded by chimerism results. **B** Retrospective molecular MRD results, quantified by qPCR with patient specific assays for *PRKCE::ETV6 and ETV6::INO80D*. Asterisks mark detectable PCR-results that are below the quantifiable range (positive, not quantifiable). Modified induction therapy (Prednisolon, Daunorubicin, Vincristin, i. th. Methotrexat), CPM: cyclophosphamide, MTX: methotrexate, modif. HR1: modif. high risk 1 block, NEL: nelarabin, VP16: etoposide, DNX-FLA: Daunoxome, Fludarabin, high dose cytarabin i. th. Methotrexat

The patient with this unique translocation is a 4-year-old boy, presented to the Department of Pediatric Hematology and Oncology of the Medical University Hamburg-Eppendorf (UKE) in 2011. Flow cytometric analysis revealed blasts which were positive for CD5, CD7, CD11b, CD34, HLADR, CD45 and cyCD3 compatible with a near ETP-ALL. Induction therapy was initiated with a modified CoALL-08–09 High Risk (HR) standard treatment [20]. Since there were no clonal markers available for molecular MRD, therapy response was monitored by cytomorphological analysis only (Fig. 3A). Since this patient was diagnosed with induction failure with central nervous system (CNS) infiltration, he was stratified to the HR intensified treatment arm and received an individual treatment based on response criteria, including high-dose cytarabine, followed by DNX-FLA (Daunorubicine, Fludarabin, and Cytarabin), a TVTG regimen (Topotecan, Vinorelbine, Thiotepa, Dexamethasone and Gemcitabine) [21] and Alemtuzumab. Ultimately, he received a bone marrow transplantation (HSCT) with an HLA-mismatched unrelated donor (HLA 9/10). The presence of complete chimerism after HSCT indicates the absence of relapse. Retrospectively, we utilized the genomic breakpoints (gBP) of *ETV6*::*INO80D* and *PRKCE*::*ETV6* as molecular MRD markers to confirm a complete molecular remission by qPCR (Fig. 3B).

### *PRKCE::ETV6 *induces factor independent cell-line growth in vitro

**Fig. 4:**
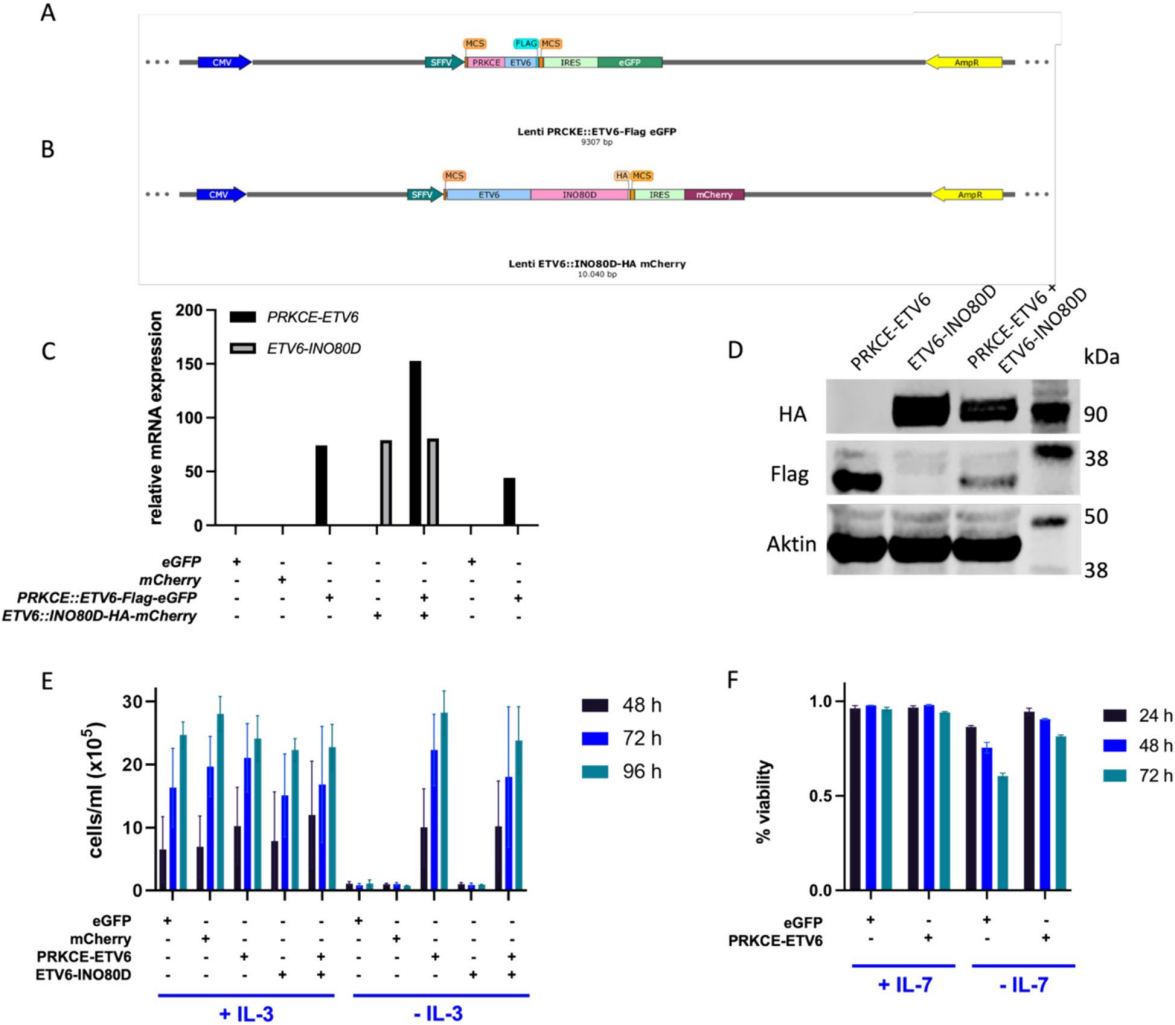
Functional characterization of PRKCE::ETV6 and ETV6::INO80D fusion proteins. **A** + **B** Maps of lentiviral plasmids containing the fusion constructs for *PRKCE::ETV6-Flag-eGFP* and *ETV6::INO80D-HA-mCherry*. Validation of fusion constructs by **C** relative quantification of mRNA level by qPCR in Ba/F3 and D1 cells and **D** Western Blot in Ba/F3. Cells were lentivirally transduced with empty vector (*eGFP* or *mCherry* appropriate to the corresponding fusion protein), *PRKCE::ETV6-Flag-eGFP*, or *ETV6::INO80D-HA-mCherry* or in combination of both, as indicated. Ct-values from qPCR *for PRKCE::ETV6* and *ETV6::INO80D* were normalized to murine *GAPDH* and are presented as 2^-ΔΔCt*1000. Fusion proteins were detected in Ba/F3 cells via HA- or Flag-Tag Western Blot. Transformation potentials of PRKCE::ETV6-Flag and ETV6::INO80D-HA were analysed by **E** IL-3 withdrawal in Ba/F3 cells and **F** IL-7 withdrawal in D1 cells. Ba/F3 were counted 48 h, 72 h, and 96 h after seeding and proliferation potential is depicted (cells/ml medium). For D1 cells, the percentage of living cells was determined after 24 h, 48 h, and 72 h. Both plots show the average values of three independent experiments with technical triplicates each

To evaluate the transforming potential of the *ETV6::INO80D* and *PRKCE::ETV6* fusions, both fusions were exogenously expressed in Ba/F3 cells upon lentiviral transduction. Vector constructs are shown in Fig. 4A and B. Successful transduction of Ba/F3 and D1 was confirmed by relative quantification of mRNA transcripts using qPCR (Fig. 4C). Full-length protein translation was confirmed by Western Blot in Ba/F3 cells (Fig. 4D). Upon withdrawal of interleukin-3 (IL-3), Ba/F3 cells expressing the PRKCE::ETV6 fusion protein continued to proliferate in the absence of IL-3, whereas cells transduced with an empty vector control and *ETV6::INO80D* did not exhibit IL-3-independent growth (Fig. 4E). Ba/F3 cells co-transduced with both fusion transcripts also showed IL-3 independent cell survival and growth, demonstrating similar behavior to Ba/F3 cells expressing only the PRKCE::ETV6 fusion protein (Fig. 4E).

For validation purposes, *PRKCE::ETV6* was then introduced into the T-cell line D1 via lentiviral transduction. D1 cells expressing the PRKCE::ETV6 fusion protein exhibited reduced apoptosis rates in the absence of interleukin-7 (IL-7) compared to those only transduced with the empty vector control (Fig. 4F*)*.

## Discussion

Several genetic alterations have previously been described to exert an influence on the pathogenesis of T-ALL [22]. Abnormalities such as aberrant expression of transcription factors or cell cycle regulators, as well as fusion genes resulting from chromosomal translocations, are frequent and lead to aberrant differentiation, proliferation and apoptosis of T-cell progenitors [23]. Here, we identified novel and recurrent gene fusion partners for commonly translocated genes in ALL with potential driving capacities by utilization of targeted gc-HTS.

The most common gene–gene fusion is *STIL::TAL1*, arising from a submicroscopic interstitial deletion between *STIL* and *TAL1*. In line with literature, we identified *STIL:TAL1* in 17% of our cases [7]. In our cohort, *STIL::TAL1* had no impact on EFS. Interestingly, we identified two *JAK2* rearrangements, the novel *CEP120::JAK2* fusion and *PCM1::JAK2*, which has so far only been reported in two T-ALL [15]. The latter was found in a case of therapy-refractory disease and nowadays, such a patient could benefit from treatment with Ruxolitinib [24].

The *ETV6* gene has been identified to play a role in the development of multiple hematologic malignancies [25]. As a member of the E26 transformation specific (ETS) family of transcription factors, ETV6 contains three major functional domains. The C-terminal ETS-domain mediates specific DNA-binding activities, while the sterile alpha motif (SAM) domain facilitates together with the internal domain protein–protein interactions with ETS-factors [26]. Various single nucleotide variations (SNVs) and chromosomal translocations involving *ETV6* have been identified that lead to leukemic development [27, 28]. While most of the ETS-domain proteins are transcriptional activators, ETV6 has been found to act as a strong transcriptional repressor [29].

Here, we found three patients with *ETV6*-fusions, namely *MN1::ETV6, ETV6::CRX*, and *PRKCE::ETV6/ETV6::INO80D*, resulting from a complex rearrangement involving chromosome 2 and 12. *MN1::ETV6* is a recurrent translocation in AML but to our knowledge, this is the first reported case in T-ALL [30]. *ETV6::CRX* has recently been described in an ETP-ALL patient, where the CRX homeodomain was postulated to induce a T-cell differentiation block [16]. *ETV6::INO80D* has been described in two ETP-ALL cases but none of these patients had the co-translocation with *PRKCE *[11]. Interestingly, all three *ETV6*-translocated cases in our cohort show a near ETP immunophenotype, consistent with the findings by Van Vlierberghe et al., who postulated a predominant occurrence of *ETV6* mutations in undifferentiated T-ALL [28]. The term “near ETP-ALL” refers to a group of T-ALL that shares a comparable genotype and phenotype as ETP-ALL but exhibits higher CD5 expression levels [16]. ETP-ALL has recently been defined by the WHO as a high-risk subtype of T-ALL, accounting for 11–12% of pediatric ALL cases [31]. Originating from early T-cell precursors, multilineage pluripotency is retained and it displays a distinct immunophenotypic and genomic profile compared to other subtypes of T-ALL.

The novel in-frame fusion *PRKCE::ETV6* results in a PRKCE::ETV6 fusion protein, containing C2-domain of PRKCE and ETS-domain of ETV6. Since the kinase domain is not contained the PRKCE::ETV6 fusion protein, we propose that the oncogenic potential of PRKCE::ETV6 fusion protein might arise from dysregulated protein–protein interaction, leading to aberrant transcriptional regulation of ETV6 target genes. The fusion was identified in a child with near ETP-ALL ultimately undergoing HSCT. Like ETP-ALL, near ETP-ALL show five-fold higher rates of induction failure compared to non-ETP T-ALL subgroups, but near ETP-ALL is not associated with an inferior survival [16]. However, Wood et al. demonstrated that near ETP-ALL patients with a day-29 MRD ≥ 0.1% exhibited reduced OS and 5-year EFS, which was not observed in patients with ETP-ALL [16]. Another study demonstrated that outcomes for these patients were improved by allogenic HSCT [32]. These findings suggest that intensified treatment strategies, including HSCT, may enhance survival rates in childhood ETP-ALL [33]. Given the high rate of induction failure, Wood et al. postulates an early therapy intensification may be necessary, targeting resistance pathways specific to ETP-ALL [16].

Since MRD is such a crucial parameter for therapy guidance, the choice of a stable molecular MRD marker is essential for an appropriate therapy regiment. Most patients are monitored via the quantification of leukemia specific *TCR* or *immunoglobulin* (*IG*) sequences by real time PCR. However, consistent with its undifferentiated phenotype, ETP-ALL and near ETP-ALL frequently lack *TCR* or *IG* gene rearrangement, which hampers the identification of molecular MRD marker [34]. Unfortunately, results from the morphological analysis reveal that significant discrepancies can occur, as demonstrated post-HSCT, where relapse of blasts was excluded by chimerism analysis. The morphological assessment indicated over 15% blasts, reflecting regenerating donor-derived cells rather than a relapse of the original malignancy, particularly as the patient has remained in remission to date. Therefore, alternative genomic markers are required to allow highly sensitive molecular MRD monitoring and genomic breakpoints resulting from chromosomal translocations are in focus of MRD researchers. For example, Hoffmann et al. presented that *ETV6::RUNX1* breakpoints represent sensitive and stable markers for MRD in ALL [35]. In this patient, the *PRKCE*::*ETV6* fusion might be a driver mutation that could be exploited as a potential marker for molecular MRD in the future. However, as we have previously demonstrated, the careful selection of an appropriate qPCR-MRD marker plays a crucial role in the accurate patient stratification [14]. Uncommon genomic breakpoints need to be evaluated carefully before utilization for MRD.

INO80D is an essential regulator for chromatin remodeling and genome integrity and we reasoned that it acts as a tumor suppressor [36]. Unexpectedly, our in vitro studies show that only *PRKCE::ETV6* single and *PRKCE::ETV6/ETV6::INO80D* double transduced Ba/F3 cells maintain proliferation capacities after IL-3 withdrawal. *ETV6::INO80D* alone did not show any transforming potential. To confirm these findings in a more relevant T-cell lineage context we transduced D1-cells as well. D1 is a murine thymocyte cell line that is IL-7-dependent for survival and growth in vitro [37]. In line with our findings in the Ba/F3 pro-B cell model, *PRKCE::ETV6* rescues D1 T-cells from cell death. Altogether, these data provide evidence that *PRKCE::ETV6* represents a biological active gene–gene fusion event that qualifies as a reliable MRD target in this near ETP-ALL.

## Conclusions

In this study, we focused on uncommon gene–gene fusion events in a comprehensive T-ALL cohort. Utilizing a targeted gc-HTS approach we were able to identify *n* = 60 gene–gene fusion events, eight of which are rare or unknown in T-ALL. Notably, a complex rearrangement identified in a near ETP-ALL, involving chr. 2 and chr. 12, resulted in the two fusion proteins INO80D::ETV6 and PRKCE::ETV6. Only PRKCE::ETV6 showed factor independence in Ba/F3 and D1 cell line models supporting the notion of *PRKCE::ETV6* as a driver oncogene in ETP-ALL. Our data underline the relevance of ETV6-fusion proteins in the development of undifferentiated T-ALL and support the use of genomic breakpoints within *ETV6* as potential MRD markers for (near) ETP-ALL that otherwise lack clonal markers.

## Methods

### Patient samples

We screened a patient cohort of 229 T-ALL, recruited for COALL03 (*n* = 89), COALL09 (*n* = 108), COALL20 (*n* = 23) and Alltogether1 (*n* = 9). Clinical information and hematological values were retrieved from patient files. The Ethics Committee of the Hamburg Medical Association gave the ethics vote PVN7286. The study was performed in accordance with the Declaration of Helsinki. Written informed consent was obtained from all patients and/or their legal representatives. All sample IDs are completely pseudonymized.

### gc-HTS

To detect gene–gene fusions in T-ALL cases we utilized our targeted genomic capture high-throughput sequencing (gc-HTS) approach. Method is described elsewhere in detail [13]. Beside the *TCR* and *immunoglobulin* loci, we capture the loci of *ETV6, KMT2A, TCF3, TAL1, ABL1/2, PDGFRb, CSF1R, EPOR,* and *JAK2*. Chromosomal coordinates from the probes are listed in Supplemental Table 1. We used genomic DNA from bone marrow (BM) or peripheral blood (PB) from day of diagnosis for the screen. Gene fusions were detected by Segemehl [13].

### RNA isolation, cDNA synthesis and qPCR

RNA was isolated from 5–10 mio. cells from with RNeasy Mini Kit (QIAGEN) and reverse transcribed to cDNA by M-MLV reverse transcription from Promega for 1 h at 37 °C using random primers (Promega), both appropriate to the manufacturer’s instructions. Quantitative qPCR was performed with SYBR Green I (Roche), according to the manufacturer’s instructions. Sanger sequencing of the qPCR products was performed with according primers at Microsynth Seqlab GmbH. Primer sequences are listed in Table 2.

**Table 2:** Overview of primers for qPCR, Sanger sequencing, and cloning

Fusion	5’ Primer	3’ Primer
*CALM2::ABL1*	ATGATCAGGGAAGCAGATATTGAT	TGAGGCTCAAAGTCAGATGC
*CEP120::JAK2*	TGGAATCTGCAACTAAGTCTAAACT	ATCCCGGTCTTCAAAGGCAC
*ETV6::CRX*	ACCACATCATGGTCTCTGTCT	CGCTCCCGCCGCTGCT
*ETV6::INO80D*	CAACCTCTCTCATCGGGAAG	CGAGAGAAGTTACAGCCTGC
*PRKCE::ETV6*	ATTGATCTGGAGCCAGAAGGAA	AGCAACTGATAGACGTAATCCC
*KMT2A::STK4*	ACCACAGGATGGAGACTACG	CTCTTGCTGATGGGGTAGGT
*MN1::ETV6*	CAGAACCCCAACAGCAAAGAA	GTGTTGCTGTCAATTGGCCTTA
*PCM1::JAK2*	CGTCTGCACAGGCCAGCCTG	GTCCTGTAGAGGGTCATACC
*SLC12A6::NUTM1*	CTTCGCTGGCAACTGTTGCA	GTTGGTGGGAGAAAGGGAAGT
*ETV6_WT*	TGACCAAAGAGGACTTTCGCTAT	GAATCCGAGGTTTCCTCTGC
*INO80D_WT*	CCAGCGCCTCTCACTCTG	AATGCCGCAGAATCAGGATGAA
*PRKCE_WT*	CATCCAGTTTGAGGAGCTGC	ACTCTTCCTTCTGGCTCCAG
*PRKCE::ETV6* full length	ATGGTAGTGTTCAATGGCCTTCT	TCAGCATTCATCTTCTTGGTATATTT (Flag-Tag: TCACTTGTCGTCATCGTCTTTGTAGTC GCATTCATCTTCTTGGTATATTT)
*ETV6::INO80D* full length	ATGTCTGAGACTCCTGCTCAG	TCAGTTAGGGGAGGGAAAGG (HA-Tag: TCAAGCGTAATCTGGAACATC GTATGGGTAGTTAGGGGAGGGAAAGG)

### Relative quantification

Relative expression levels were depicted after normalization to beta-2-microglobulin (B2M; human) or mGAPDH (murine) as a reference gene (2^-ΔΔCt*1000). Mononuclear cells from peripheral blood (PBMCs) from healthy donors were used as normal controls.

### Molecular MRD quantification

Quantification of gBP as molecular MRD markers followed EUROMRD guidelines [38]. Primer and probes are P::E-fw CAGGATGGCTCAGATCAGATTTA, P::E-rv GCTCAGATCAGATTTAGGGAACA, P::E-TM ACGTTTGATGCTCTCTGCCTTGCA, E::I-fw TCAAGTTTTCCTGGAAAACAGC, E::I-rv TTGAGCACAACACTAAATGCC, E::I-TM TGGTATTGCCTATGTCTGCTTCCATGC.

### Cloning of full-length transcripts

The fusion transcripts were amplified by using the TOPO XL-2 complete PCR Cloning Kit, with One Shot™ OmniMAX™ 2 T1^R^ Chemically Competent *E. coli* Cells (Thermo Fisher Scientific) and full-length primer sets (Table 2). 3’ Flag-tag to *PRKCE::ETV6* and 3’ HA-tag to *ETV6::INO80D* were added by PCR with indicated primers (Table 2). Tagged full length constructs were subcloned into pLenti-LeGO-eGFP or -mCherry, using T4 DNA ligase (Thermo Fisher Scientific).

### Cell culture

Human embryonic kidney 293 T (HEK293T) cells were cultured in DMEM (gibco) supplemented with 10% FBS (gibco), 2% HEPES Buffer Solution (1 M) (gibco), 1% sodium pyruvate (100 mM) (gibco). Ba/F3 cells were cultured 1 × 10^5^/ml in RPMI 1640 medium (gibco) supplemented with 10% heat inactivated (h.i.) FBS (gibco) and 10 ng/ml recombinant murine interleukin-3 (PeproTech). D1 cells were cultured 5 × 10^5^/ml in RPMI 1640 medium (gibco) supplemented with 10% h.i. FBS (gibco), 2% HEPES Buffer Solution (1 M) (gibco) and 25 ng/ml recombinant murine IL-7 (biolegend). All cells were incubated at 37 °C and 5% CO_2._

### Lentiviral transduction

Lentiviral transduction was described in detail elsewhere [39]. Successful transduction of Ba/F3 and D1 cells was verified 48 h later with fluorescence activated cell sorting (FACS). Two weeks post-transduction eGFP-/mCherry positive cells were purified by FACS.

### Proliferation and viability assays

For long-term viability and proliferation experiments transduced cells were seeded without interleukin supplementation as triplicates in 24-well plates. Ba/F3 cells were seeded at a concentration of 1 × 10^5^/ml and D1 cells at a density of 5 × 10^5^/ml. Cell counts and viability of Ba/F3 and D1 cells were assessed on day 2, day 3, and day 4 using Trypan Blue and Countess 3 (Thermo Fisher Scientific). Results represent average values of three independent replicates.

### Western blot analysis

Cells were lysed with Ripa Lysis Buffer (ChemCruz) complemented with PMSF, sodium orthovanadate and protease inhibitor cocktail (all from Santa Cruz) and sonicated with the Bioruptor™ (Diagenode). The samples were loaded on NuPAGE™ 4–12% Bis–Tris Gel (Thermo Fisher Scientific) with 1X MES SDS Running Buffer (Thermo Fisher Scientific, NuPAGE®, 20X). Protein gels were transferred as a wet-blot at 100 V (equals to 200 mA), using Towbin-Buffer (6.06 g Tris, 28.8 g Glycine, 400 ml Methanol, 1600 ml H_2_O), Whatman paper and Nitrocellulose Blotting Membrane (GE Healthcare, Amersham™ Protran™ 0.45 µm NC). Following antibodies were used: 1:500 monoclonal Anti Flag-tag antibody (Lot: #VC296117, Thermos Fisher Scientific), 1:1000 Anti HA-Tag monoclonal antibody (Lot: #ZF393513, Thermo Fisher Scientific), 1:5000 Monoclonal Anti-ß-Actin (Lot: 067M4856V, Sigma), 1:20.000 IRDye® 800CW Goat anti-rabbit IgG (Lot: #D30425-15, Li-Cor), 1:20.000 IRDye® 680RD Goat anti-mouse IgG (Lot: #D30418-05, Li-Cor). Images were acquired and analyzed using Odyssey XF Imager (Li-Cor).

## Supplementary Information

Supplementary Material 1.

Supplementary Material 2.

Supplementary Material 3.

## Data Availability

Additional data that support the findings of this study are available from the corresponding author.

## Abbreviations

ALL: Acute lymphoblastic leukemia
AML: Acute myeloid leukemia
BCP-ALL: B-cell precursor-ALL
CNS: Central nervous system
ETP-ALL: Early T-cell precursor acute lymphoblastic leukemia
EFS: Event-free survival rate
ETS: E26 transformation specific
HSCT: Hematopoietic stem cell transplantation
IDR: Intrinsically disordered region
IG/TR: Immunoglobulin/T-cell receptor
IL-3: Interleukin-3
IL-7: Interleukin-7
MRD: Minimal residual disease
OS: Overall survival
SAM: Sterile alpha motif
SNV: Single nucleotide variation
T-ALL: T-cell acute lymphoblastic leukemia
TCR: T-cell receptor
UTR: Untranslated region
