# Supplementary material for: Characterization of a PRKCE::ETV6 fusion as a potential oncogenic driver in T-cell acute lymphoblastic leukemia
Source: Mol Cell Pediatr. 2025 Oct 22;12:16. doi: 10.1186/s40348-025-00208-x (PMC12546167; doi:10.1186/s40348-025-00208-x)
Supplement: Supplementary file 1 — Supplementary Material 1. [file 40348_2025_208_MOESM1_ESM.docx]

Supplemental Data

**Title: Characterization of a *PRKCE*::*ETV6* fusion as a potential oncogenic driver in T cell acute lymphoblastic leukemia**

Authors:

Esther L. Monsees^a,b^, Udo zur Stadt^a^, Julia Strauss^b^, Sabrina Schuster^b^, Nadja Kleist^a^, Richard T. Hauch^a^, Michael Spohn^b^, Gerrit Wolters-Eisfeld^b^, Martin A. Horstmann^a,b^, Gabriele Escherich^a^ and Lena Behrmann^a^

^a^Clinic of Pediatric Hematology and Oncology, University Medical Center Hamburg-Eppendorf, Hamburg, Germany

^b^Research Institute Children's Cancer Center Hamburg, Hamburg, Germany

Corresponding Author: Lena Behrmann, [le.behrmann@uke.de](mailto:le.behrmann@uke.de)

**Supplemental Table 1: List of chromosomal regions captured for gc-HTS.** List provides information about the panel, i. a. gene names, chromosomal localizations and length of the sites in basepairs (bp). Ko represents control genes for each chromosome and are used for quality control. BED format of the list is available from corresponding author.
