# Supplementary material for: Characterization of a PRKCE::ETV6 fusion as a potential oncogenic driver in T-cell acute lymphoblastic leukemia
Source: Mol Cell Pediatr. 2025 Oct 22;12:16. doi: 10.1186/s40348-025-00208-x (PMC12546167; doi:10.1186/s40348-025-00208-x)
Supplement: Supplementary file 2 — Supplementary Material 2. [file 40348_2025_208_MOESM2_ESM.pptx]

## Slide 1
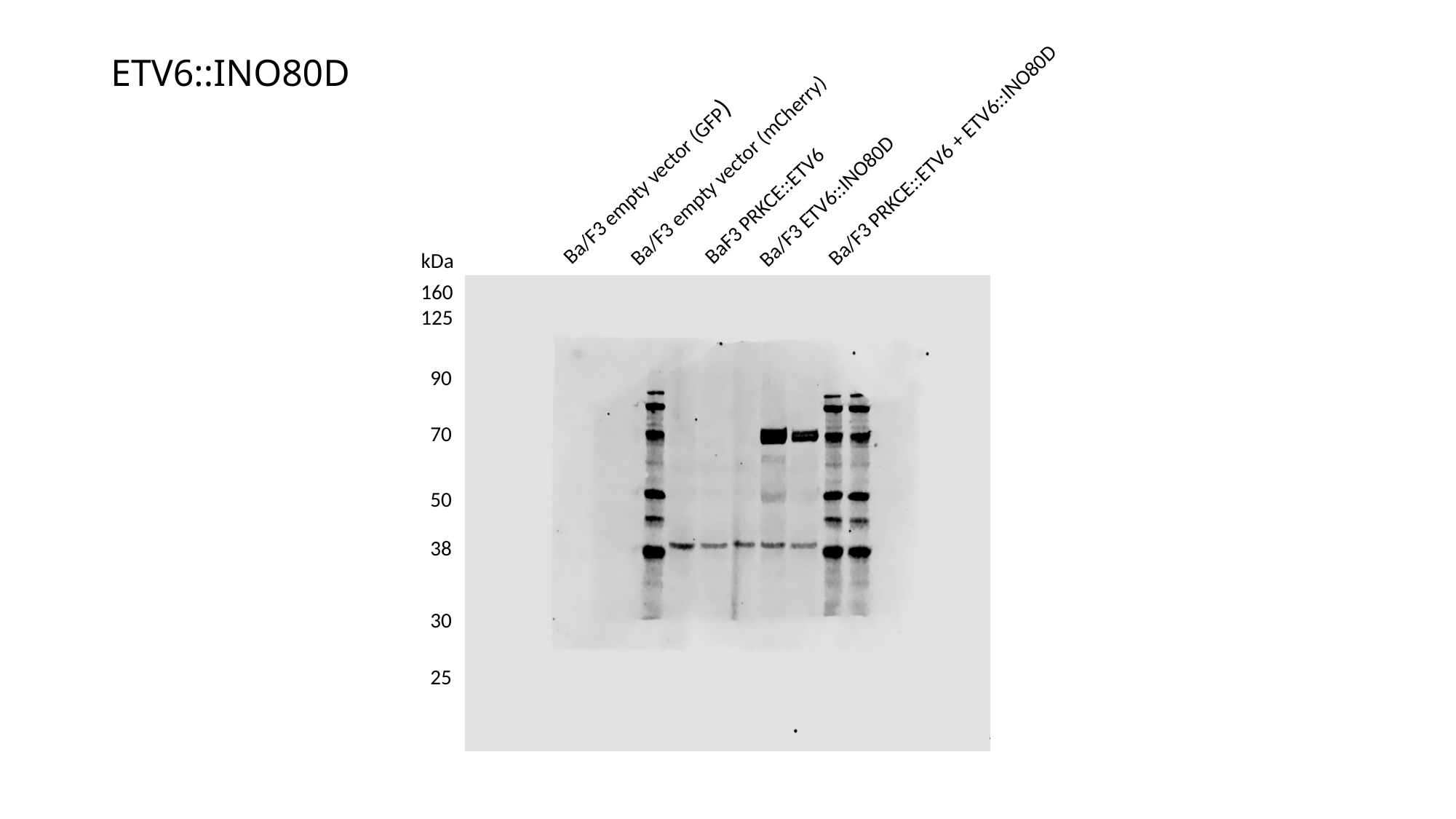

# ETV6::INO80D
Ba/F3 PRKCE::ETV6 + ETV6::INO80D
Ba/F3 empty vector (mCherry)
Ba/F3 empty vector (GFP)
Ba/F3 ETV6::INO80D
BaF3 PRKCE::ETV6
kDa
160
125
 90
 70
 50
 38
 30
 25

## Slide 2
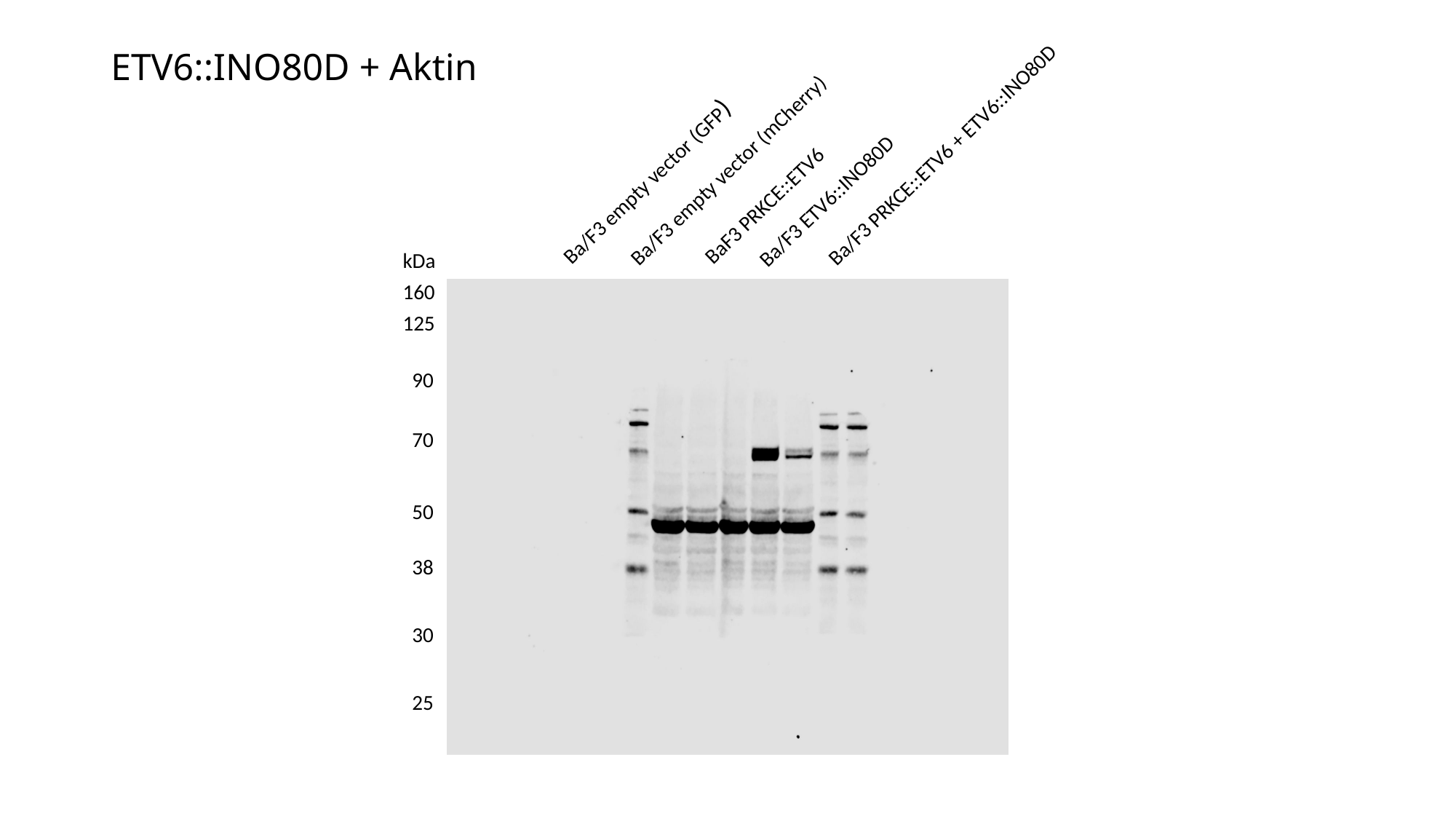

ETV6::INO80D + Aktin
Ba/F3 PRKCE::ETV6 + ETV6::INO80D
Ba/F3 empty vector (mCherry)
Ba/F3 empty vector (GFP)
Ba/F3 ETV6::INO80D
BaF3 PRKCE::ETV6
kDa
160
125
 90
 70
 50
 38
 30
 25

## Slide 3
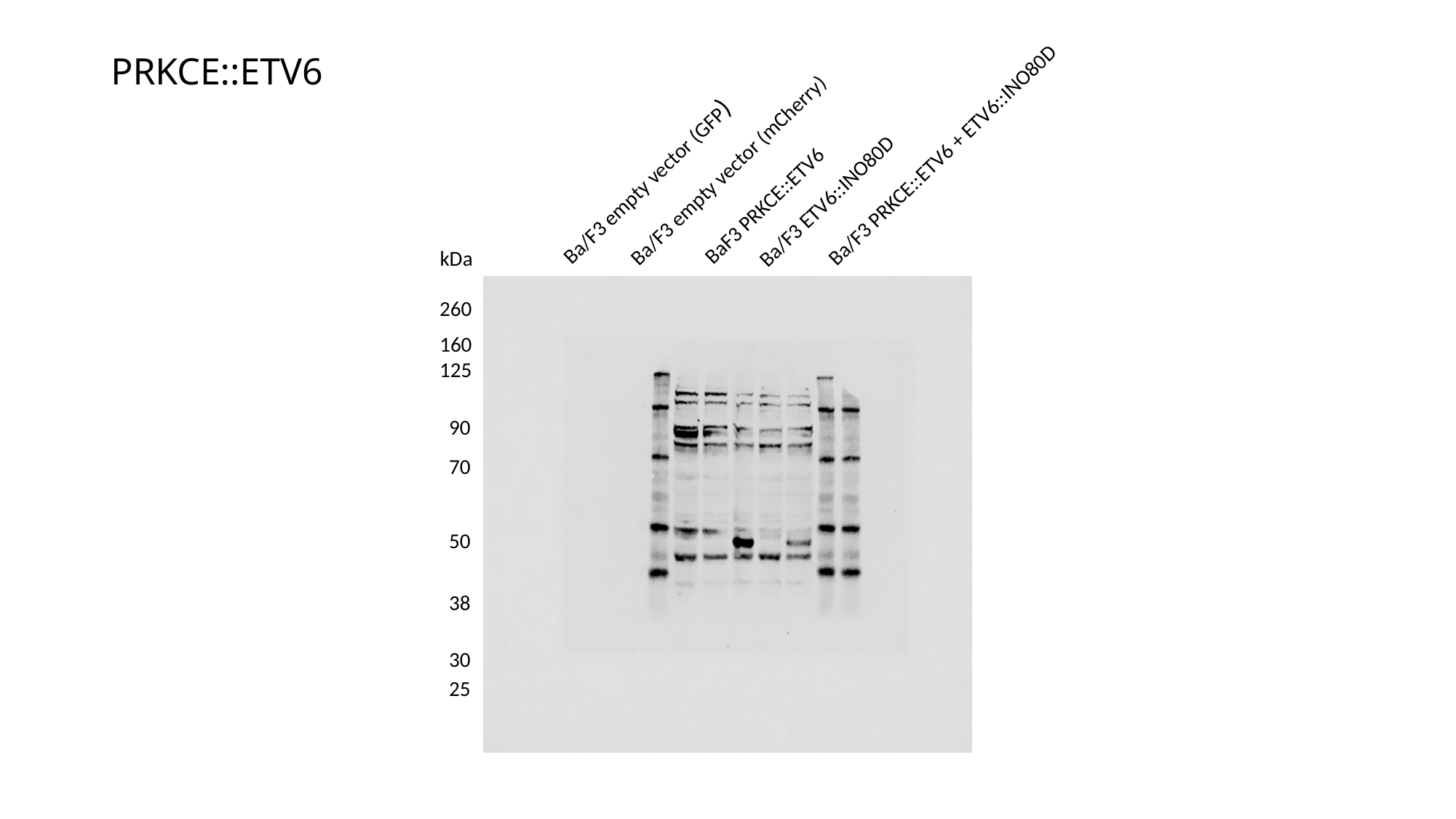

PRKCE::ETV6
Ba/F3 PRKCE::ETV6 + ETV6::INO80D
Ba/F3 empty vector (mCherry)
Ba/F3 empty vector (GFP)
Ba/F3 ETV6::INO80D
BaF3 PRKCE::ETV6
kDa
260
160
125
 90
 70
 50
 38
 30
 25

## Slide 4
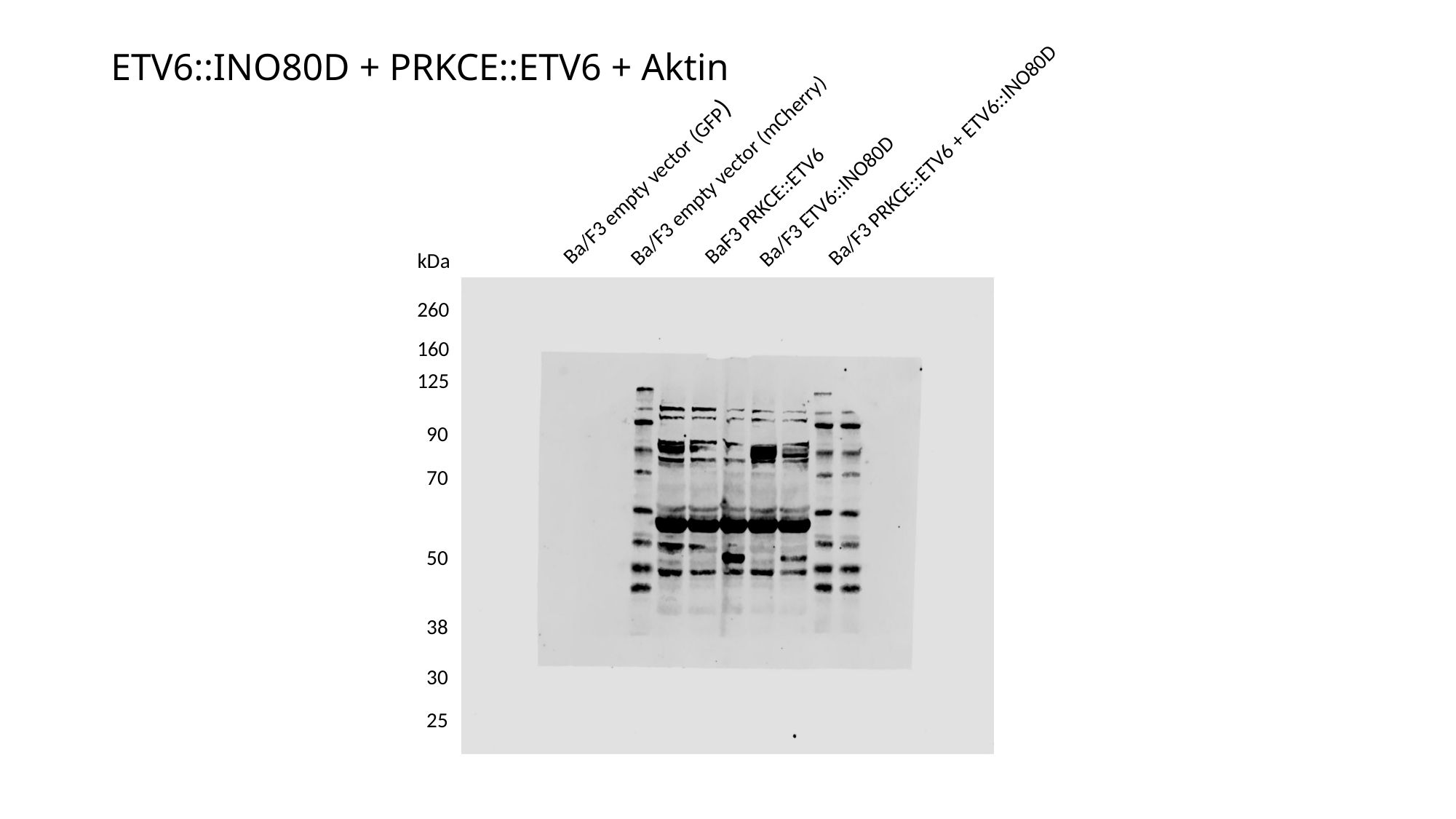

ETV6::INO80D + PRKCE::ETV6 + Aktin
Ba/F3 PRKCE::ETV6 + ETV6::INO80D
Ba/F3 empty vector (mCherry)
Ba/F3 empty vector (GFP)
Ba/F3 ETV6::INO80D
BaF3 PRKCE::ETV6
kDa
260
160
125
 90
 70
 50
 38
 30
 25
